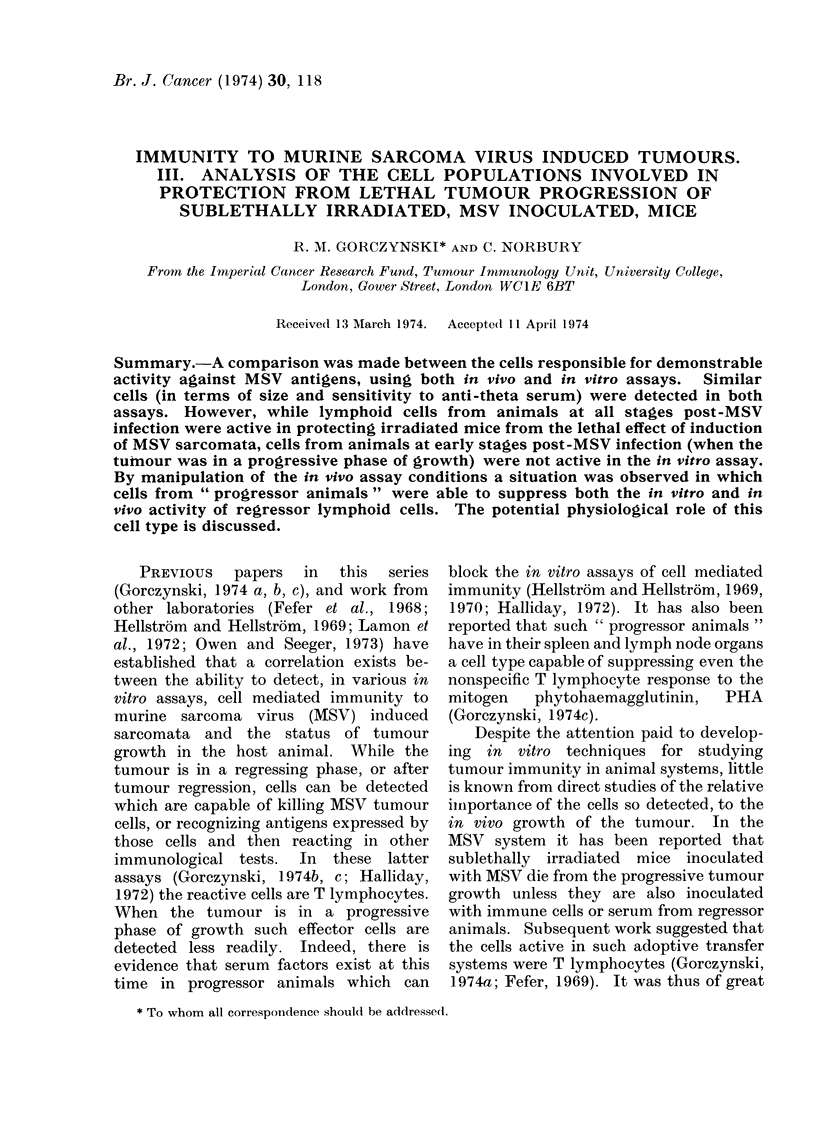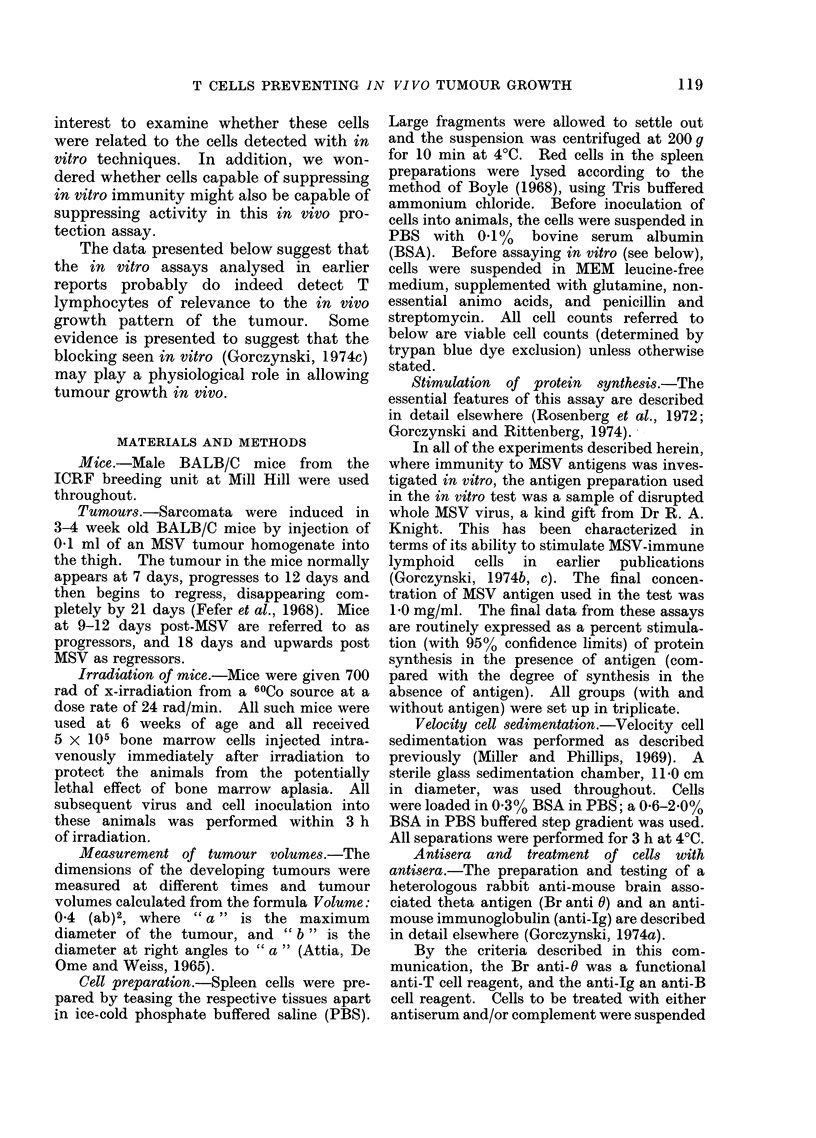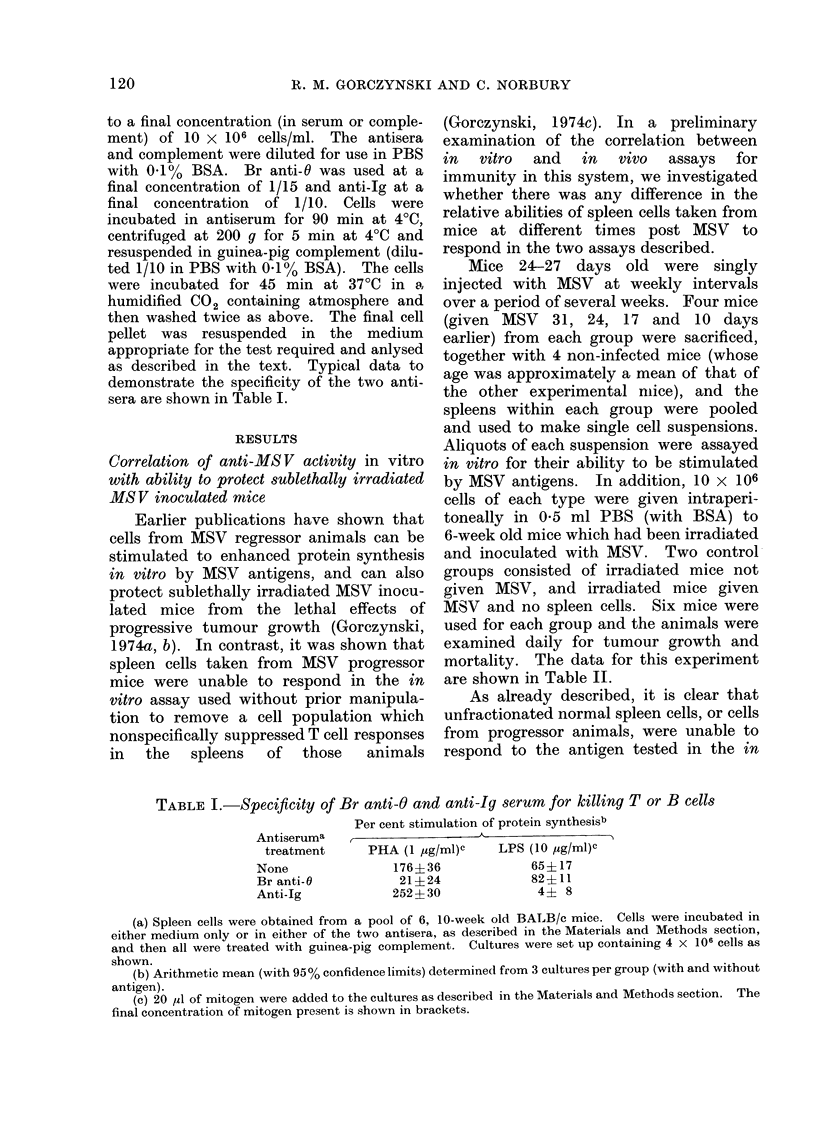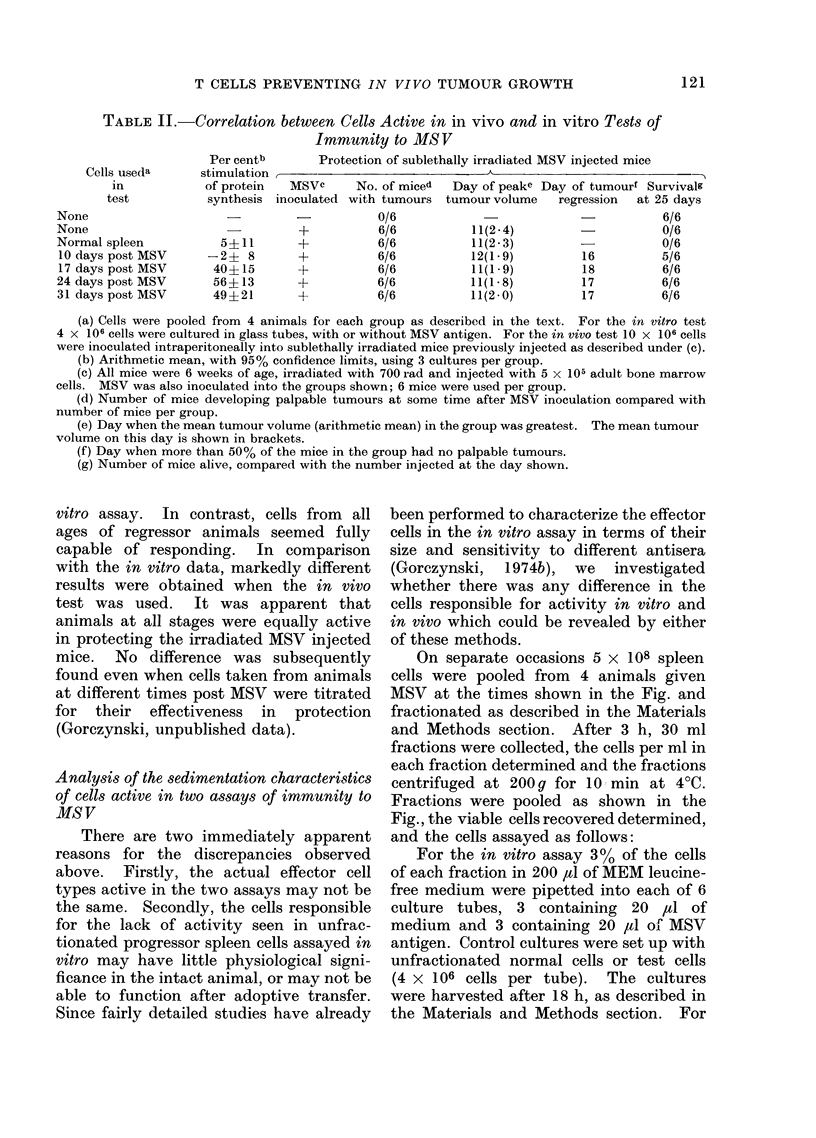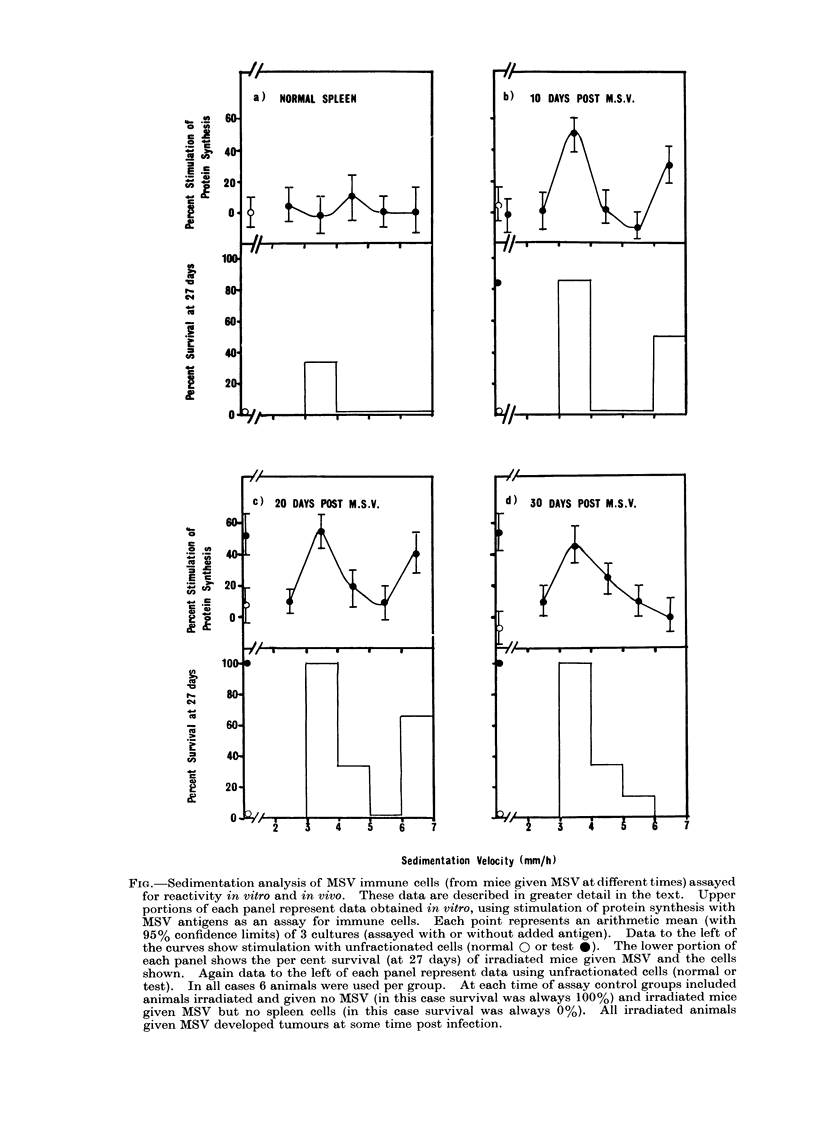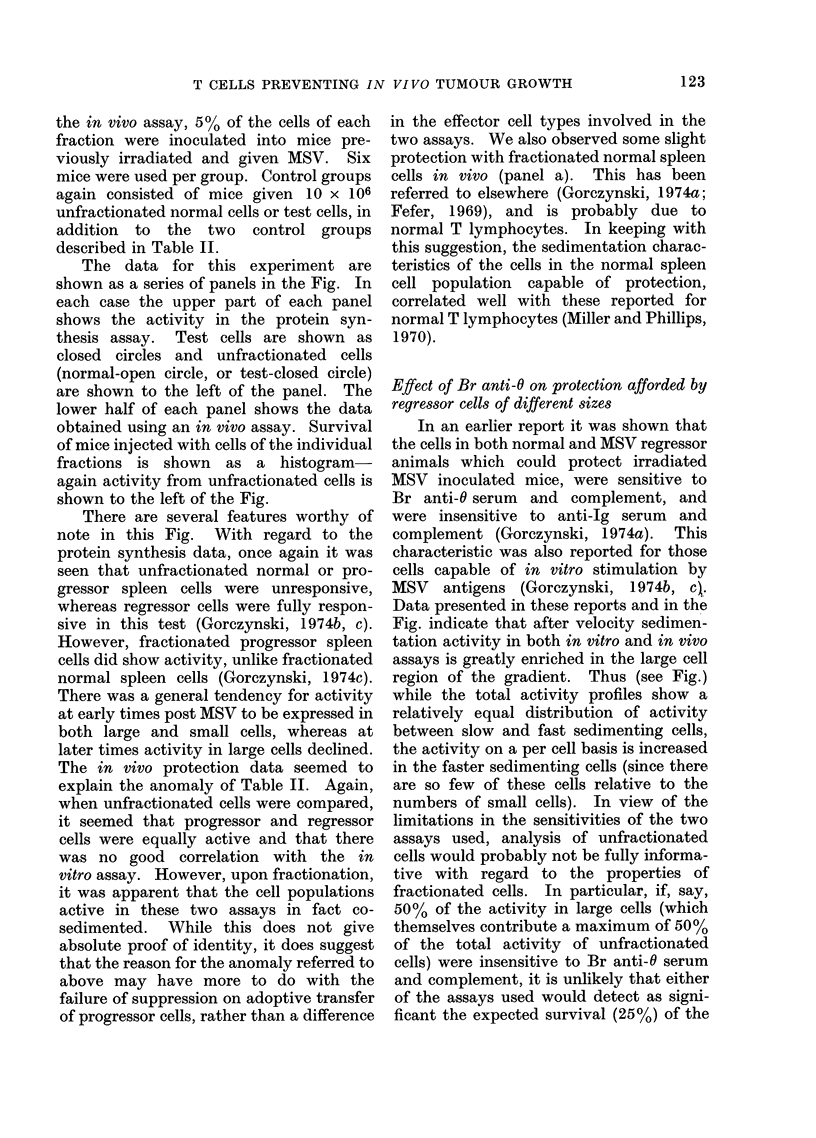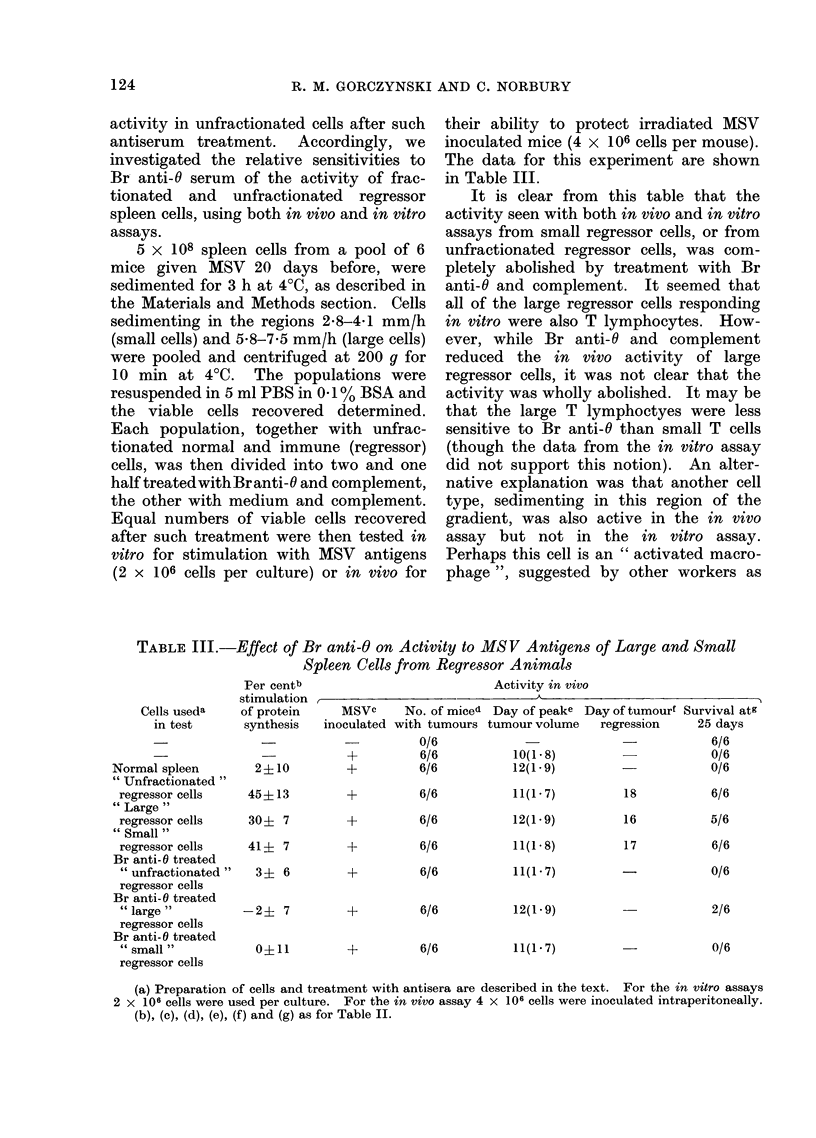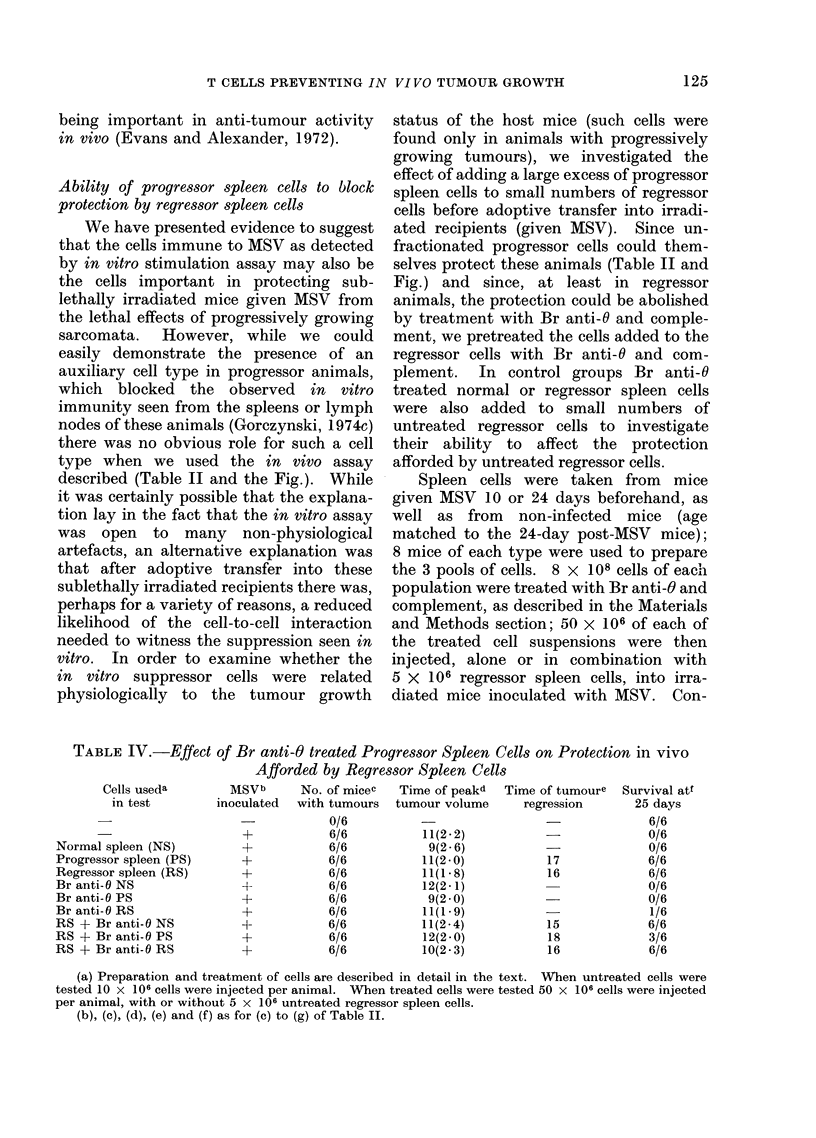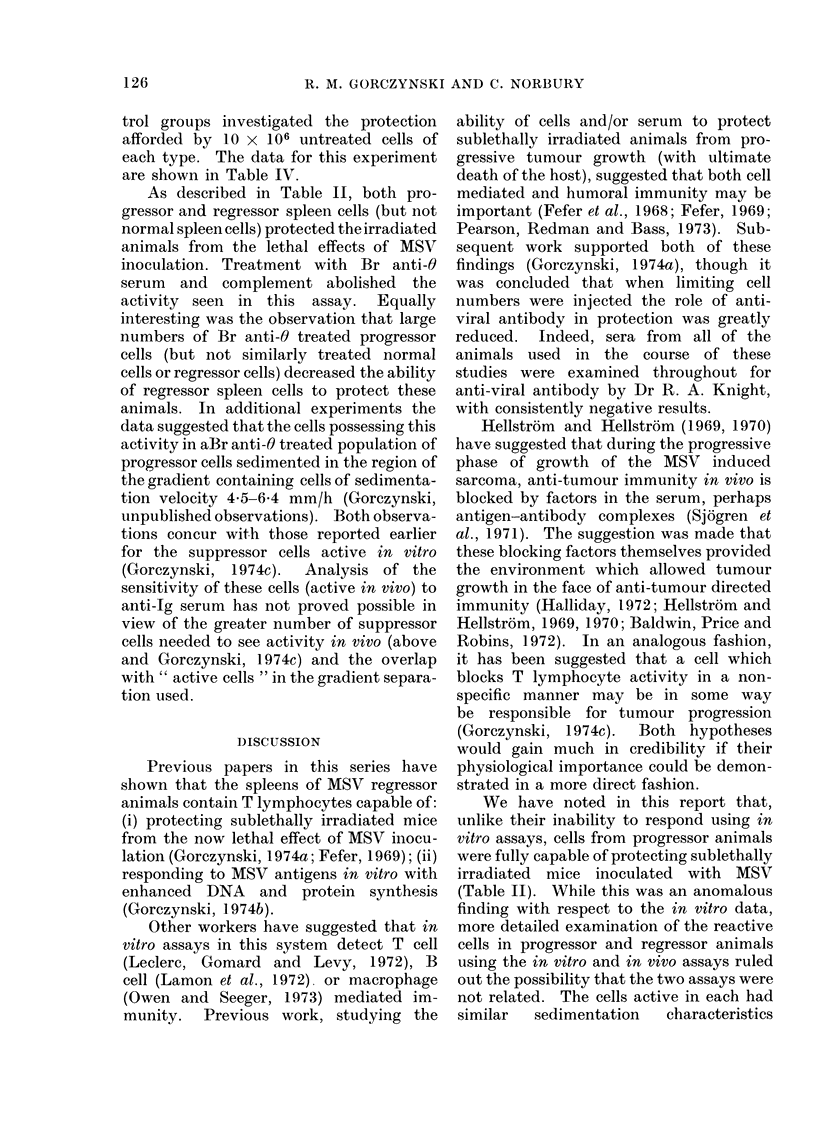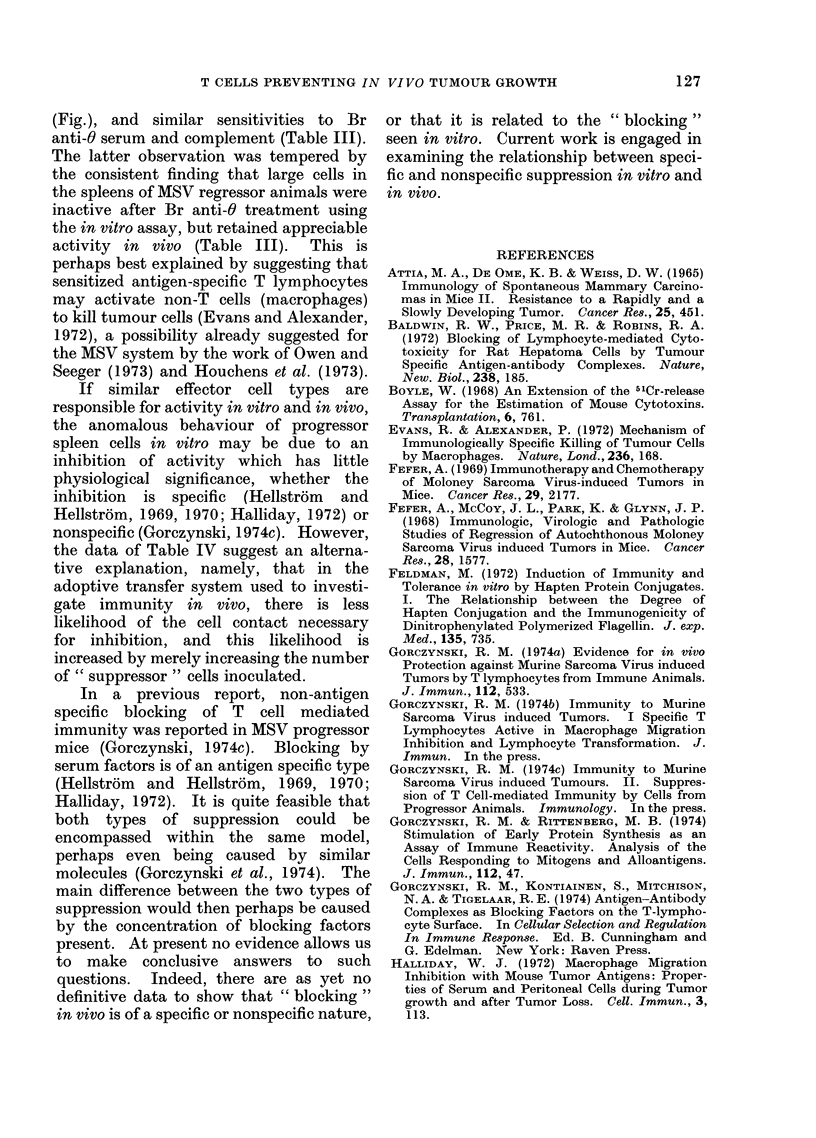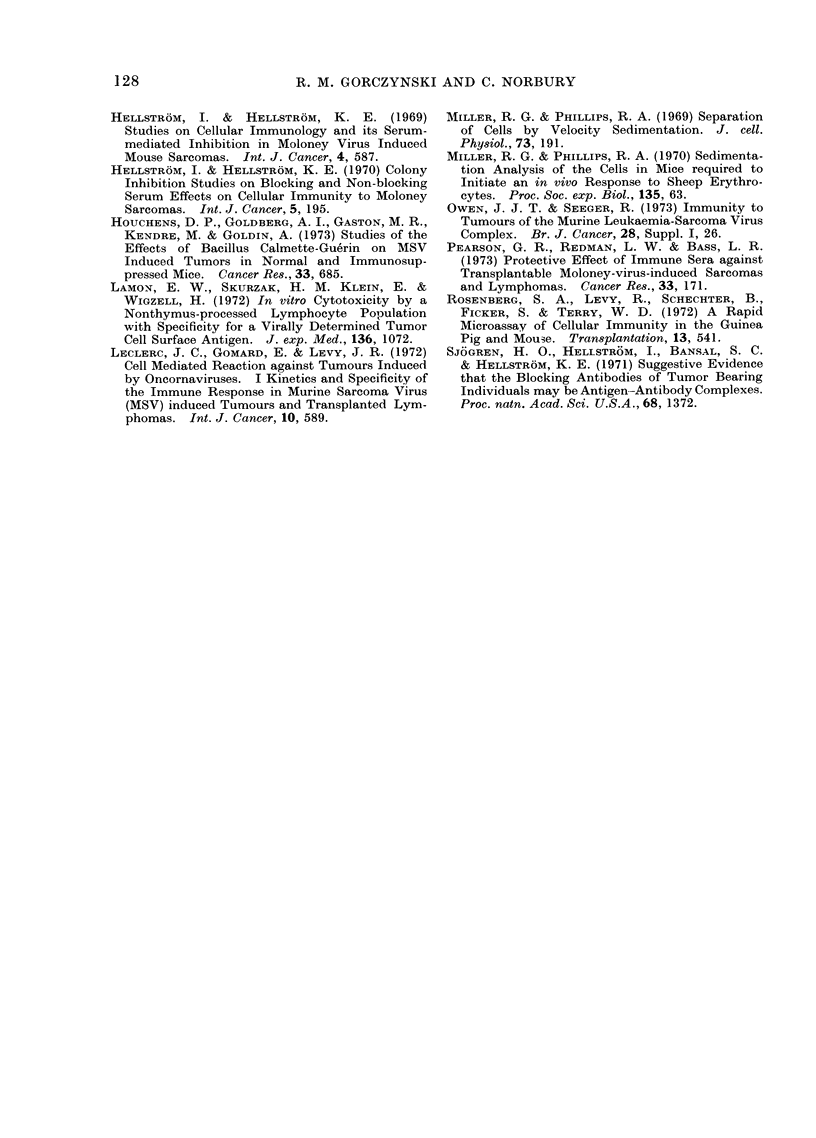# Immunity to Murine Sarcoma Virus Induced Tumors. III. Analysis of the Cell Populations Involved in Protection from Lethal Tumour Progression of Sublethally Irradiated, MSV Inoculated, Mice

**DOI:** 10.1038/bjc.1974.122

**Published:** 1974-08

**Authors:** R. M. Gorczynski, C. Norbury

## Abstract

A comparison was made between the cells responsible for demonstrable activity against MSV antigens, using both *in vivo* and *in vitro* assays. Similar cells (in terms of size and sensitivity to anti-theta serum) were detected in both assays. However, while lymphoid cells from animals at all stages post-MSV infection were active in protecting irradiated mice from the lethal effect of induction of MSV sarcomata, cells from animals at early stages post-MSV infection (when the tumour was in a progressive phase of growth) were not active in the *in vitro* assay. By manipulation of the *in vivo* assay conditions a situation was observed in which cells from “progressor animals” were able to suppress both the *in vitro* and *in vivo* activity of regressor lymphoid cells. The potential physiological role of this cell type is disussed.


*Br. J. Cancer* (1974) **30**, 118

### IMMUNITY TO MURINE SARCOMA VIRUS INDUCED TUMOURS. III. ANALYSIS OF THE CELL POPULATIONS INVOLVED IN PROTECTION FROM LETHAL TUMOUR PROGRESSION OF SUBLETHALLY IRRADIATED, MSV INOCULATED, MICE

R. M. GORCZYNSKI* AND C. NORBURY

*From the Imperial Cancer Research Fund, Tumour Immunology Unit, University College, London, Gower Street, London WC1E 6BT*

Received 13 March 1974. Accepted 11 April 1974

**Summary.—A comparison was made between the cells responsible for demonstrable activity against MSV antigens, using both *in vivo* and *in vitro* assays. Similar cells (in terms of size and sensitivity to anti-theta serum) were detected in both assays. However, while lymphoid cells from animals at all stages post-MSV infection were active in protecting irradiated mice from the lethal effect of induction of MSV sarcomata, cells from animals at early stages post-MSV infection (when the tumour was in a progressive phase of growth) were not active in the *in vitro* assay. By manipulation of the *in vivo* assay conditions a situation was observed in which cells from " progressor animals " were able to suppress both the *in vitro* and *in vivo* activity of regressor lymphoid cells. The potential physiological role of this cell type is discussed.**

PREVIOUS papers in this series (Gorczynski, 1974 *a*, *b*, *c*), and work from other laboratories (Fefer *et al.*, 1968; Hellström and Hellström, 1969; Lamon *et al.*, 1972; Owen and Seeger, 1973) have established that a correlation exists between the ability to detect, in various *in vitro* assays, cell mediated immunity to murine sarcoma virus (MSV) induced sarcomata and the status of tumour growth in the host animal. While the tumour is in a regressing phase, or after tumour regression, cells can be detected which are capable of killing MSV tumour cells, or recognizing antigens expressed by those cells and then reacting in other immunological tests. In these latter assays (Gorczynski, 1974*b*, *c*; Halliday, 1972) the reactive cells are T lymphocytes. When the tumour is in a progressive phase of growth such effector cells are detected less readily. Indeed, there is evidence that serum factors exist at this time in progressor animals which can block the *in vitro* assays of cell mediated immunity (Hellström and Hellström, 1969, 1970; Halliday, 1972). It has also been reported that such " progressor animals " have in their spleen and lymph node organs a cell type capable of suppressing even the nonspecific T lymphocyte response to the mitogen phytohaemagglutinin, PHA (Gorczynski, 1974*c*).

Despite the attention paid to developing *in vitro* techniques for studying tumour immunity in animal systems, little is known from direct studies of the relative importance of the cells so detected, to the *in vivo* growth of the tumour. In the MSV system it has been reported that sublethally irradiated mice inoculated with MSV die from the progressive tumour growth unless they are also inoculated with immune cells or serum from regressor animals. Subsequent work suggested that the cells active in such adoptive transfer systems were T lymphocytes (Gorczynski, 1974*a*; Fefer, 1969). It was thus of great

* To whom all correspondence should be addressed.

interest to examine whether these cells were related to the cells detected with *in vitro* techniques. In addition, we wondered whether cells capable of suppressing *in vitro* immunity might also be capable of suppressing activity in this *in vivo* protection assay.

The data presented below suggest that the *in vitro* assays analysed in earlier reports probably do indeed detect T lymphocytes of relevance to the *in vivo* growth pattern of the tumour. Some evidence is presented to suggest that the blocking seen *in vitro* (Gorczynski, 1974*c*) may play a physiological role in allowing tumour growth *in vivo*.

### MATERIALS AND METHODS

*Mice*.—Male BALB/C mice from the ICRF breeding unit at Mill Hill were used throughout.

*Tumours*.—Sarcomata were induced in 3–4 week old BALB/C mice by injection of 0·1 ml of an MSV tumour homogenate into the thigh. The tumour in the mice normally appears at 7 days, progresses to 12 days and then begins to regress, disappearing completely by 21 days (Fefer *et al.*, 1968). Mice at 9–12 days post-MSV are referred to as progressors, and 18 days and upwards post MSV as regressors.

*Irradiation of mice*.—Mice were given 700 rad of x-irradiation from a $^{60}$Co source at a dose rate of 24 rad/min. All such mice were used at 6 weeks of age and all received $5 \times 10^5$ bone marrow cells injected intravenously immediately after irradiation to protect the animals from the potentially lethal effect of bone marrow aplasia. All subsequent virus and cell inoculation into these animals was performed within 3 h of irradiation.

*Measurement of tumour volumes*.—The dimensions of the developing tumours were measured at different times and tumour volumes calculated from the formula *Volume:* 0·4 $(ab)^2$, where "$a$" is the maximum diameter of the tumour, and "$b$" is the diameter at right angles to "$a$" (Attia, De Ome and Weiss, 1965).

*Cell preparation*.—Spleen cells were prepared by teasing the respective tissues apart in ice-cold phosphate buffered saline (PBS). Large fragments were allowed to settle out and the suspension was centrifuged at 200 *g* for 10 min at 4°C. Red cells in the spleen preparations were lysed according to the method of Boyle (1968), using Tris buffered ammonium chloride. Before inoculation of cells into animals, the cells were suspended in PBS with 0·1% bovine serum albumin (BSA). Before assaying *in vitro* (see below), cells were suspended in MEM leucine-free medium, supplemented with glutamine, non-essential animo acids, and penicillin and streptomycin. All cell counts referred to below are viable cell counts (determined by trypan blue dye exclusion) unless otherwise stated.

*Stimulation of protein synthesis*.—The essential features of this assay are described in detail elsewhere (Rosenberg *et al.*, 1972; Gorczynski and Rittenberg, 1974).

In all of the experiments described herein, where immunity to MSV antigens was investigated *in vitro*, the antigen preparation used in the *in vitro* test was a sample of disrupted whole MSV virus, a kind gift from Dr R. A. Knight. This has been characterized in terms of its ability to stimulate MSV-immune lymphoid cells in earlier publications (Gorczynski, 1974*b*, *c*). The final concentration of MSV antigen used in the test was 1·0 mg/ml. The final data from these assays are routinely expressed as a percent stimulation (with 95% confidence limits) of protein synthesis in the presence of antigen (compared with the degree of synthesis in the absence of antigen). All groups (with and without antigen) were set up in triplicate.

*Velocity cell sedimentation*.—Velocity cell sedimentation was performed as described previously (Miller and Phillips, 1969). A sterile glass sedimentation chamber, 11·0 cm in diameter, was used throughout. Cells were loaded in 0·3% BSA in PBS; a 0·6–2·0% BSA in PBS buffered step gradient was used. All separations were performed for 3 h at 4°C.

*Antisera and treatment of cells with antisera*.—The preparation and testing of a heterologous rabbit anti-mouse brain associated theta antigen (Br anti $\theta$) and an anti-mouse immunoglobulin (anti-Ig) are described in detail elsewhere (Gorczynski, 1974*a*).

By the criteria described in this communication, the Br anti-$\theta$ was a functional anti-T cell reagent, and the anti-Ig an anti-B cell reagent. Cells to be treated with either antiserum and/or complement were suspended to a final concentration (in serum or complement) of $10 \times 10^6$ cells/ml. The antisera and complement were diluted for use in PBS with 0·1% BSA. Br anti-$\theta$ was used at a final concentration of 1/15 and anti-Ig at a final concentration of 1/10. Cells were incubated in antiserum for 90 min at 4°C, centrifuged at 200 *g* for 5 min at 4°C and resuspended in guinea-pig complement (diluted 1/10 in PBS with 0·1% BSA). The cells were incubated for 45 min at 37°C in a humidified $CO_2$ containing atmosphere and then washed twice as above. The final cell pellet was resuspended in the medium appropriate for the test required and anlysed as described in the text. Typical data to demonstrate the specificity of the two antisera are shown in Table I.

### RESULTS

### *Correlation of anti-MSV activity* in vitro *with ability to protect sublethally irradiated MSV inoculated mice*

Earlier publications have shown that cells from MSV regressor animals can be stimulated to enhanced protein synthesis *in vitro* by MSV antigens, and can also protect sublethally irradiated MSV inoculated mice from the lethal effects of progressive tumour growth (Gorczynski, 1974*a*, *b*). In contrast, it was shown that spleen cells taken from MSV progressor mice were unable to respond in the *in vitro* assay used without prior manipulation to remove a cell population which nonspecifically suppressed T cell responses in the spleens of those animals (Gorczynski, 1974*c*). In a preliminary examination of the correlation between *in vitro* and *in vivo* assays for immunity in this system, we investigated whether there was any difference in the relative abilities of spleen cells taken from mice at different times post MSV to respond in the two assays described.

Mice 24–27 days old were singly injected with MSV at weekly intervals over a period of several weeks. Four mice (given MSV 31, 24, 17 and 10 days earlier) from each group were sacrificed, together with 4 non-infected mice (whose age was approximately a mean of that of the other experimental mice), and the spleens within each group were pooled and used to make single cell suspensions. Aliquots of each suspension were assayed *in vitro* for their ability to be stimulated by MSV antigens. In addition, $10 \times 10^6$ cells of each type were given intraperitoneally in 0·5 ml PBS (with BSA) to 6-week old mice which had been irradiated and inoculated with MSV. Two control groups consisted of irradiated mice not given MSV, and irradiated mice given MSV and no spleen cells. Six mice were used for each group and the animals were examined daily for tumour growth and mortality. The data for this experiment are shown in Table II.

As already described, it is clear that unfractionated normal spleen cells, or cells from progressor animals, were unable to respond to the antigen tested in the *in*

TABLE I.—*Specificity of Br anti-θ and anti-Ig serum for killing T or B cells*

| Antiserum[a] treatment | Per cent stimulation of protein synthesis[b] PHA (1 μg/ml)[c] | LPS (10 μg/ml)[c] |
|---|---|---|
| None | 176±36 | 65±17 |
| Br anti-θ | 21±24 | 82±11 |
| Anti-Ig | 252±30 | 4± 8 |

(a) Spleen cells were obtained from a pool of 6, 10-week old BALB/c mice. Cells were incubated in either medium only or in either of the two antisera, as described in the Materials and Methods section, and then all were treated with guinea-pig complement. Cultures were set up containing $4 \times 10^6$ cells as shown.

(b) Arithmetic mean (with 95% confidence limits) determined from 3 cultures per group (with and without antigen).

(c) 20 μl of mitogen were added to the cultures as described in the Materials and Methods section. The final concentration of mitogen present is shown in brackets.

TABLE II.—*Correlation between Cells Active in* in vivo *and* in vitro *Tests of Immunity to MSV*

| Cells used[a] in test | Per cent[b] stimulation of protein synthesis | Protection of sublethally irradiated MSV injected mice | | | | |
|---|---|---|---|---|---|---|
| | | MSV[c] inoculated | No. of mice[d] with tumours | Day of peak[e] tumour volume | Day of tumour[f] regression | Survival[g] at 25 days |
| None | — | — | 0/6 | — | — | 6/6 |
| None | — | + | 6/6 | 11(2·4) | — | 0/6 |
| Normal spleen | 5±11 | + | 6/6 | 11(2·3) | — | 0/6 |
| 10 days post MSV | −2± 8 | + | 6/6 | 12(1·9) | 16 | 5/6 |
| 17 days post MSV | 40±15 | + | 6/6 | 11(1·9) | 18 | 6/6 |
| 24 days post MSV | 56±13 | + | 6/6 | 11(1·8) | 17 | 6/6 |
| 31 days post MSV | 49±21 | + | 6/6 | 11(2·0) | 17 | 6/6 |

(a) Cells were pooled from 4 animals for each group as described in the text. For the *in vitro* test $4 \times 10^6$ cells were cultured in glass tubes, with or without MSV antigen. For the *in vivo* test $10 \times 10^6$ cells were inoculated intraperitoneally into sublethally irradiated mice previously injected as described under (c).

(b) Arithmetic mean, with 95% confidence limits, using 3 cultures per group.

(c) All mice were 6 weeks of age, irradiated with 700 rad and injected with $5 \times 10^5$ adult bone marrow cells. MSV was also inoculated into the groups shown; 6 mice were used per group.

(d) Number of mice developing palpable tumours at some time after MSV inoculation compared with number of mice per group.

(e) Day when the mean tumour volume (arithmetic mean) in the group was greatest. The mean tumour volume on this day is shown in brackets.

(f) Day when more than 50% of the mice in the group had no palpable tumours.

(g) Number of mice alive, compared with the number injected at the day shown.

*vitro* assay. In contrast, cells from all ages of regressor animals seemed fully capable of responding. In comparison with the *in vitro* data, markedly different results were obtained when the *in vivo* test was used. It was apparent that animals at all stages were equally active in protecting the irradiated MSV injected mice. No difference was subsequently found even when cells taken from animals at different times post MSV were titrated for their effectiveness in protection (Gorczynski, unpublished data).

### *Analysis of the sedimentation characteristics of cells active in two assays of immunity to MSV*

There are two immediately apparent reasons for the discrepancies observed above. Firstly, the actual effector cell types active in the two assays may not be the same. Secondly, the cells responsible for the lack of activity seen in unfractionated progressor spleen cells assayed *in vitro* may have little physiological significance in the intact animal, or may not be able to function after adoptive transfer. Since fairly detailed studies have already been performed to characterize the effector cells in the *in vitro* assay in terms of their size and sensitivity to different antisera (Gorczynski, 1974*b*), we investigated whether there was any difference in the cells responsible for activity *in vitro* and *in vivo* which could be revealed by either of these methods.

On separate occasions $5 \times 10^8$ spleen cells were pooled from 4 animals given MSV at the times shown in the Fig. and fractionated as described in the Materials and Methods section. After 3 h, 30 ml fractions were collected, the cells per ml in each fraction determined and the fractions centrifuged at 200*g* for 10 min at 4°C. Fractions were pooled as shown in the Fig., the viable cells recovered determined, and the cells assayed as follows:

For the *in vitro* assay 3% of the cells of each fraction in 200 μl of MEM leucine-free medium were pipetted into each of 6 culture tubes, 3 containing 20 μl of medium and 3 containing 20 μl of MSV antigen. Control cultures were set up with unfractionated normal cells or test cells ($4 \times 10^6$ cells per tube). The cultures were harvested after 18 h, as described in the Materials and Methods section. For

FIG.—Sedimentation analysis of MSV immune cells (from mice given MSV at different times) assayed for reactivity *in vitro* and *in vivo*. These data are described in greater detail in the text. Upper portions of each panel represent data obtained *in vitro*, using stimulation of protein synthesis with MSV antigens as an assay for immune cells. Each point represents an arithmetic mean (with 95% confidence limits) of 3 cultures (assayed with or without added antigen). Data to the left of the curves show stimulation with unfractionated cells (normal ○ or test ●). The lower portion of each panel shows the per cent survival (at 27 days) of irradiated mice given MSV and the cells shown. Again data to the left of each panel represent data using unfractionated cells (normal or test). In all cases 6 animals were used per group. At each time of assay control groups included animals irradiated and given no MSV (in this case survival was always 100%) and irradiated mice given MSV but no spleen cells (in this case survival was always 0%). All irradiated animals given MSV developed tumours at some time post infection.

the *in vivo* assay, 5% of the cells of each fraction were inoculated into mice previously irradiated and given MSV. Six mice were used per group. Control groups again consisted of mice given $10 \times 10^6$ unfractionated normal cells or test cells, in addition to the two control groups described in Table II.

The data for this experiment are shown as a series of panels in the Fig. In each case the upper part of each panel shows the activity in the protein synthesis assay. Test cells are shown as closed circles and unfractionated cells (normal-open circle, or test-closed circle) are shown to the left of the panel. The lower half of each panel shows the data obtained using an *in vivo* assay. Survival of mice injected with cells of the individual fractions is shown as a histogram—again activity from unfractionated cells is shown to the left of the Fig.

There are several features worthy of note in this Fig. With regard to the protein synthesis data, once again it was seen that unfractionated normal or progressor spleen cells were unresponsive, whereas regressor cells were fully responsive in this test (Gorczynski, 1974*b*, *c*). However, fractionated progressor spleen cells did show activity, unlike fractionated normal spleen cells (Gorczynski, 1974*c*). There was a general tendency for activity at early times post MSV to be expressed in both large and small cells, whereas at later times activity in large cells declined. The *in vivo* protection data seemed to explain the anomaly of Table II. Again, when unfractionated cells were compared, it seemed that progressor and regressor cells were equally active and that there was no good correlation with the *in vitro* assay. However, upon fractionation, it was apparent that the cell populations active in these two assays in fact cosedimented. While this does not give absolute proof of identity, it does suggest that the reason for the anomaly referred to above may have more to do with the failure of suppression on adoptive transfer of progressor cells, rather than a difference in the effector cell types involved in the two assays. We also observed some slight protection with fractionated normal spleen cells *in vivo* (panel a). This has been referred to elsewhere (Gorczynski, 1974*a*; Fefer, 1969), and is probably due to normal T lymphocytes. In keeping with this suggestion, the sedimentation characteristics of the cells in the normal spleen cell population capable of protection, correlated well with these reported for normal T lymphocytes (Miller and Phillips, 1970).

*Effect of Br anti-θ on protection afforded by regressor cells of different sizes*

In an earlier report it was shown that the cells in both normal and MSV regressor animals which could protect irradiated MSV inoculated mice, were sensitive to Br anti-θ serum and complement, and were insensitive to anti-Ig serum and complement (Gorczynski, 1974*a*). This characteristic was also reported for those cells capable of *in vitro* stimulation by MSV antigens (Gorczynski, 1974*b*, *c*). Data presented in these reports and in the Fig. indicate that after velocity sedimentation activity in both *in vitro* and *in vivo* assays is greatly enriched in the large cell region of the gradient. Thus (see Fig.) while the total activity profiles show a relatively equal distribution of activity between slow and fast sedimenting cells, the activity on a per cell basis is increased in the faster sedimenting cells (since there are so few of these cells relative to the numbers of small cells). In view of the limitations in the sensitivities of the two assays used, analysis of unfractionated cells would probably not be fully informative with regard to the properties of fractionated cells. In particular, if, say, 50% of the activity in large cells (which themselves contribute a maximum of 50% of the total activity of unfractionated cells) were insensitive to Br anti-θ serum and complement, it is unlikely that either of the assays used would detect as significant the expected survival (25%) of the activity in unfractionated cells after such antiserum treatment. Accordingly, we investigated the relative sensitivities to Br anti-$\theta$ serum of the activity of fractionated and unfractionated regressor spleen cells, using both *in vivo* and *in vitro* assays.

$5 \times 10^8$ spleen cells from a pool of 6 mice given MSV 20 days before, were sedimented for 3 h at 4°C, as described in the Materials and Methods section. Cells sedimenting in the regions 2·8–4·1 mm/h (small cells) and 5·8–7·5 mm/h (large cells) were pooled and centrifuged at 200 *g* for 10 min at 4°C. The populations were resuspended in 5 ml PBS in 0·1% BSA and the viable cells recovered determined. Each population, together with unfractionated normal and immune (regressor) cells, was then divided into two and one half treated with Br anti-$\theta$ and complement, the other with medium and complement. Equal numbers of viable cells recovered after such treatment were then tested *in vitro* for stimulation with MSV antigens ($2 \times 10^6$ cells per culture) or *in vivo* for their ability to protect irradiated MSV inoculated mice ($4 \times 10^6$ cells per mouse). The data for this experiment are shown in Table III.

It is clear from this table that the activity seen with both *in vivo* and *in vitro* assays from small regressor cells, or from unfractionated regressor cells, was completely abolished by treatment with Br anti-$\theta$ and complement. It seemed that all of the large regressor cells responding *in vitro* were also T lymphocytes. However, while Br anti-$\theta$ and complement reduced the *in vivo* activity of large regressor cells, it was not clear that the activity was wholly abolished. It may be that the large T lymphoctyes were less sensitive to Br anti-$\theta$ than small T cells (though the data from the *in vitro* assay did not support this notion). An alternative explanation was that another cell type, sedimenting in this region of the gradient, was also active in the *in vivo* assay but not in the *in vitro* assay. Perhaps this cell is an " activated macrophage ", suggested by other workers as

TABLE III.—*Effect of Br anti-$\theta$ on Activity to MSV Antigens of Large and Small Spleen Cells from Regressor Animals*

| Cells used[a] in test | Per cent[b] stimulation of protein synthesis | Activity *in vivo*: MSV[c] inoculated | No. of mice[d] with tumours | Day of peak[e] tumour volume | Day of tumour[f] regression | Survival at[g] 25 days |
|---|---|---|---|---|---|---|
| — | — | — | 0/6 | — | — | 6/6 |
| — | — | + | 6/6 | 10(1·8) | — | 0/6 |
| Normal spleen | 2±10 | + | 6/6 | 12(1·9) | — | 0/6 |
| " Unfractionated " regressor cells | 45±13 | + | 6/6 | 11(1·7) | 18 | 6/6 |
| " Large " regressor cells | 30± 7 | + | 6/6 | 12(1·9) | 16 | 5/6 |
| " Small " regressor cells | 41± 7 | + | 6/6 | 11(1·8) | 17 | 6/6 |
| Br anti-$\theta$ treated " unfractionated " regressor cells | 3± 6 | + | 6/6 | 11(1·7) | — | 0/6 |
| Br anti-$\theta$ treated " large " regressor cells | −2± 7 | + | 6/6 | 12(1·9) | — | 2/6 |
| Br anti-$\theta$ treated " small " regressor cells | 0±11 | + | 6/6 | 11(1·7) | — | 0/6 |

(a) Preparation of cells and treatment with antisera are described in the text. For the *in vitro* assays $2 \times 10^6$ cells were used per culture. For the *in vivo* assay $4 \times 10^6$ cells were inoculated intraperitoneally.
(b), (c), (d), (e), (f) and (g) as for Table II.

being important in anti-tumour activity *in vivo* (Evans and Alexander, 1972).

*Ability of progressor spleen cells to block protection by regressor spleen cells*

We have presented evidence to suggest that the cells immune to MSV as detected by *in vitro* stimulation assay may also be the cells important in protecting sublethally irradiated mice given MSV from the lethal effects of progressively growing sarcomata. However, while we could easily demonstrate the presence of an auxiliary cell type in progressor animals, which blocked the observed *in vitro* immunity seen from the spleens or lymph nodes of these animals (Gorczynski, 1974*c*) there was no obvious role for such a cell type when we used the *in vivo* assay described (Table II and the Fig.). While it was certainly possible that the explanation lay in the fact that the *in vitro* assay was open to many non-physiological artefacts, an alternative explanation was that after adoptive transfer into these sublethally irradiated recipients there was, perhaps for a variety of reasons, a reduced likelihood of the cell-to-cell interaction needed to witness the suppression seen *in vitro*. In order to examine whether the *in vitro* suppressor cells were related physiologically to the tumour growth status of the host mice (such cells were found only in animals with progressively growing tumours), we investigated the effect of adding a large excess of progressor spleen cells to small numbers of regressor cells before adoptive transfer into irradiated recipients (given MSV). Since unfractionated progressor cells could themselves protect these animals (Table II and Fig.) and since, at least in regressor animals, the protection could be abolished by treatment with Br anti-$\theta$ and complement, we pretreated the cells added to the regressor cells with Br anti-$\theta$ and complement. In control groups Br anti-$\theta$ treated normal or regressor spleen cells were also added to small numbers of untreated regressor cells to investigate their ability to affect the protection afforded by untreated regressor cells.

Spleen cells were taken from mice given MSV 10 or 24 days beforehand, as well as from non-infected mice (age matched to the 24-day post-MSV mice); 8 mice of each type were used to prepare the 3 pools of cells. $8 \times 10^8$ cells of each population were treated with Br anti-$\theta$ and complement, as described in the Materials and Methods section; $50 \times 10^6$ of each of the treated cell suspensions were then injected, alone or in combination with $5 \times 10^6$ regressor spleen cells, into irradiated mice inoculated with MSV. Con-

TABLE IV.—*Effect of Br anti-θ treated Progressor Spleen Cells on Protection* in vivo *Afforded by Regressor Spleen Cells*

| Cells used[a] in test | MSV[b] inoculated | No. of mice[c] with tumours | Time of peak[d] tumour volume | Time of tumour[e] regression | Survival at[f] 25 days |
|---|---|---|---|---|---|
| — | — | 0/6 | — | — | 6/6 |
| — | + | 6/6 | 11(2·2) | — | 0/6 |
| Normal spleen (NS) | + | 6/6 | 9(2·6) | — | 0/6 |
| Progressor spleen (PS) | + | 6/6 | 11(2·0) | 17 | 6/6 |
| Regressor spleen (RS) | + | 6/6 | 11(1·8) | 16 | 6/6 |
| Br anti-θ NS | + | 6/6 | 12(2·1) | — | 0/6 |
| Br anti-θ PS | + | 6/6 | 9(2·0) | — | 0/6 |
| Br anti-θ RS | + | 6/6 | 11(1·9) | — | 1/6 |
| RS + Br anti-θ NS | + | 6/6 | 11(2·4) | 15 | 6/6 |
| RS + Br anti-θ PS | + | 6/6 | 12(2·0) | 18 | 3/6 |
| RS + Br anti-θ RS | + | 6/6 | 10(2·3) | 16 | 6/6 |

(a) Preparation and treatment of cells are described in detail in the text. When untreated cells were tested $10 \times 10^6$ cells were injected per animal. When treated cells were tested $50 \times 10^6$ cells were injected per animal, with or without $5 \times 10^6$ untreated regressor spleen cells.

(b), (c), (d), (e) and (f) as for (c) to (g) of Table II.

trol groups investigated the protection afforded by $10 \times 10^6$ untreated cells of each type. The data for this experiment are shown in Table IV.

As described in Table II, both progressor and regressor spleen cells (but not normal spleen cells) protected the irradiated animals from the lethal effects of MSV inoculation. Treatment with Br anti-$\theta$ serum and complement abolished the activity seen in this assay. Equally interesting was the observation that large numbers of Br anti-$\theta$ treated progressor cells (but not similarly treated normal cells or regressor cells) decreased the ability of regressor spleen cells to protect these animals. In additional experiments the data suggested that the cells possessing this activity in aBr anti-$\theta$ treated population of progressor cells sedimented in the region of the gradient containing cells of sedimentation velocity 4·5–6·4 mm/h (Gorczynski, unpublished observations). Both observations concur with those reported earlier for the suppressor cells active *in vitro* (Gorczynski, 1974*c*). Analysis of the sensitivity of these cells (active *in vivo*) to anti-Ig serum has not proved possible in view of the greater number of suppressor cells needed to see activity *in vivo* (above and Gorczynski, 1974*c*) and the overlap with " active cells " in the gradient separation used.

### DISCUSSION

Previous papers in this series have shown that the spleens of MSV regressor animals contain T lymphocytes capable of: (i) protecting sublethally irradiated mice from the now lethal effect of MSV inoculation (Gorczynski, 1974*a*; Fefer, 1969); (ii) responding to MSV antigens *in vitro* with enhanced DNA and protein synthesis (Gorczynski, 1974*b*).

Other workers have suggested that *in vitro* assays in this system detect T cell (Leclerc, Gomard and Levy, 1972), B cell (Lamon *et al.*, 1972), or macrophage (Owen and Seeger, 1973) mediated immunity. Previous work, studying the ability of cells and/or serum to protect sublethally irradiated animals from progressive tumour growth (with ultimate death of the host), suggested that both cell mediated and humoral immunity may be important (Fefer *et al.*, 1968; Fefer, 1969; Pearson, Redman and Bass, 1973). Subsequent work supported both of these findings (Gorczynski, 1974*a*), though it was concluded that when limiting cell numbers were injected the role of antiviral antibody in protection was greatly reduced. Indeed, sera from all of the animals used in the course of these studies were examined throughout for anti-viral antibody by Dr R. A. Knight, with consistently negative results.

Hellström and Hellström (1969, 1970) have suggested that during the progressive phase of growth of the MSV induced sarcoma, anti-tumour immunity *in vivo* is blocked by factors in the serum, perhaps antigen–antibody complexes (Sjögren *et al.*, 1971). The suggestion was made that these blocking factors themselves provided the environment which allowed tumour growth in the face of anti-tumour directed immunity (Halliday, 1972; Hellström and Hellström, 1969, 1970; Baldwin, Price and Robins, 1972). In an analogous fashion, it has been suggested that a cell which blocks T lymphocyte activity in a nonspecific manner may be in some way be responsible for tumour progression (Gorczynski, 1974*c*). Both hypotheses would gain much in credibility if their physiological importance could be demonstrated in a more direct fashion.

We have noted in this report that, unlike their inability to respond using *in vitro* assays, cells from progressor animals were fully capable of protecting sublethally irradiated mice inoculated with MSV (Table II). While this was an anomalous finding with respect to the *in vitro* data, more detailed examination of the reactive cells in progressor and regressor animals using the *in vitro* and *in vivo* assays ruled out the possibility that the two assays were not related. The cells active in each had similar sedimentation characteristics

(Fig.), and similar sensitivities to Br anti-$\theta$ serum and complement (Table III). The latter observation was tempered by the consistent finding that large cells in the spleens of MSV regressor animals were inactive after Br anti-$\theta$ treatment using the *in vitro* assay, but retained appreciable activity *in vivo* (Table III). This is perhaps best explained by suggesting that sensitized antigen-specific T lymphocytes may activate non-T cells (macrophages) to kill tumour cells (Evans and Alexander, 1972), a possibility already suggested for the MSV system by the work of Owen and Seeger (1973) and Houchens *et al.* (1973).

If similar effector cell types are responsible for activity *in vitro* and *in vivo*, the anomalous behaviour of progressor spleen cells *in vitro* may be due to an inhibition of activity which has little physiological significance, whether the inhibition is specific (Hellström and Hellström, 1969, 1970; Halliday, 1972) or nonspecific (Gorczynski, 1974*c*). However, the data of Table IV suggest an alternative explanation, namely, that in the adoptive transfer system used to investigate immunity *in vivo*, there is less likelihood of the cell contact necessary for inhibition, and this likelihood is increased by merely increasing the number of " suppressor " cells inoculated.

In a previous report, non-antigen specific blocking of T cell mediated immunity was reported in MSV progressor mice (Gorczynski, 1974*c*). Blocking by serum factors is of an antigen specific type (Hellström and Hellström, 1969, 1970; Halliday, 1972). It is quite feasible that both types of suppression could be encompassed within the same model, perhaps even being caused by similar molecules (Gorczynski *et al.*, 1974). The main difference between the two types of suppression would then perhaps be caused by the concentration of blocking factors present. At present no evidence allows us to make conclusive answers to such questions. Indeed, there are as yet no definitive data to show that " blocking " *in vivo* is of a specific or nonspecific nature, or that it is related to the " blocking " seen *in vitro*. Current work is engaged in examining the relationship between specific and nonspecific suppression *in vitro* and *in vivo*.

### REFERENCES

ATTIA, M. A., DE OME, K. B. & WEISS, D. W. (1965) Immunology of Spontaneous Mammary Carcinomas in Mice II. Resistance to a Rapidly and a Slowly Developing Tumor. *Cancer Res.*, **25**, 451.

BALDWIN, R. W., PRICE, M. R. & ROBINS, R. A. (1972) Blocking of Lymphocyte-mediated Cytotoxicity for Rat Hepatoma Cells by Tumour Specific Antigen-antibody Complexes. *Nature, New. Biol.*, **238**, 185.

BOYLE, W. (1968) An Extension of the $^{51}$Cr-release Assay for the Estimation of Mouse Cytotoxins. *Transplantation*, **6**, 761.

EVANS, R. & ALEXANDER, P. (1972) Mechanism of Immunologically Specific Killing of Tumour Cells by Macrophages. *Nature, Lond.*, **236**, 168.

FEFER, A. (1969) Immunotherapy and Chemotherapy of Moloney Sarcoma Virus-induced Tumors in Mice. *Cancer Res.*, **29**, 2177.

FEFER, A., MCCOY, J. L., PARK, K. & GLYNN, J. P. (1968) Immunologic, Virologic and Pathologic Studies of Regression of Autochthonous Moloney Sarcoma Virus induced Tumors in Mice. *Cancer Res.*, **28**, 1577.

FELDMAN, M. (1972) Induction of Immunity and Tolerance *in vitro* by Hapten Protein Conjugates. I. The Relationship between the Degree of Hapten Conjugation and the Immunogenicity of Dinitrophenylated Polymerized Flagellin. *J. exp. Med.*, **135**, 735.

GORCZYNSKI, R. M. (1974*a*) Evidence for *in vivo* Protection against Murine Sarcoma Virus induced Tumors by T lymphocytes from Immune Animals. *J. Immun.*, **112**, 533.

GORCZYNSKI, R. M. (1974*b*) Immunity to Murine Sarcoma Virus induced Tumors. I Specific T Lymphocytes Active in Macrophage Migration Inhibition and Lymphocyte Transformation. *J. Immun.* In the press.

GORCZYNSKI, R. M. (1974*c*) Immunity to Murine Sarcoma Virus induced Tumours. II. Suppression of T Cell-mediated Immunity by Cells from Progressor Animals. *Immunology*. In the press.

GORCZYNSKI, R. M. & RITTENBERG, M. B. (1974) Stimulation of Early Protein Synthesis as an Assay of Immune Reactivity. Analysis of the Cells Responding to Mitogens and Alloantigens. *J. Immun.*, **112**, 47.

GORCZYNSKI, R. M., KONTIAINEN, S., MITCHISON, N. A. & TIGELAAR, R. E. (1974) Antigen–Antibody Complexes as Blocking Factors on the T-lymphocyte Surface. In *Cellular Selection and Regulation In Immune Response*. Ed. B. Cunningham and G. Edelman. New York: Raven Press.

HALLIDAY, W. J. (1972) Macrophage Migration Inhibition with Mouse Tumor Antigens: Properties of Serum and Peritoneal Cells during Tumor growth and after Tumor Loss. *Cell. Immun.*, **3**, 113.

HELLSTRÖM, I. & HELLSTRÖM, K. E. (1969) Studies on Cellular Immunology and its Serum-mediated Inhibition in Moloney Virus Induced Mouse Sarcomas. *Int. J. Cancer*, **4**, 587.

HELLSTRÖM, I. & HELLSTRÖM, K. E. (1970) Colony Inhibition Studies on Blocking and Non-blocking Serum Effects on Cellular Immunity to Moloney Sarcomas. *Int. J. Cancer*, **5**, 195.

HOUCHENS, D. P., GOLDBERG, A. I., GASTON, M. R., KENDRE, M. & GOLDIN, A. (1973) Studies of the Effects of Bacillus Calmette-Guérin on MSV Induced Tumors in Normal and Immunosuppressed Mice. *Cancer Res.*, **33**, 685.

LAMON, E. W., SKURZAK, H. M. KLEIN, E. & WIGZELL, H. (1972) *In vitro* Cytotoxicity by a Nonthymus-processed Lymphocyte Population with Specificity for a Virally Determined Tumor Cell Surface Antigen. *J. exp. Med.*, **136**, 1072.

LECLERC, J. C., GOMARD, E. & LEVY, J. R. (1972) Cell Mediated Reaction against Tumours Induced by Oncornaviruses. I Kinetics and Specificity of the Immune Response in Murine Sarcoma Virus (MSV) induced Tumours and Transplanted Lymphomas. *Int. J. Cancer*, **10**, 589.

MILLER, R. G. & PHILLIPS, R. A. (1969) Separation of Cells by Velocity Sedimentation. *J. cell. Physiol.*, **73**, 191.

MILLER, R. G. & PHILLIPS, R. A. (1970) Sedimentation Analysis of the Cells in Mice required to Initiate an *in vivo* Response to Sheep Erythrocytes. *Proc. Soc. exp. Biol.*, **135**, 63.

OWEN, J. J. T. & SEEGER, R. (1973) Immunity to Tumours of the Murine Leukaemia-Sarcoma Virus Complex. *Br. J. Cancer*, **28**, Suppl. I, 26.

PEARSON, G. R., REDMAN, L. W. & BASS, L. R. (1973) Protective Effect of Immune Sera against Transplantable Moloney-virus-induced Sarcomas and Lymphomas. *Cancer Res.*, **33**, 171.

ROSENBERG, S. A., LEVY, R., SCHECHTER, B., FICKER, S. & TERRY, W. D. (1972) A Rapid Microassay of Cellular Immunity in the Guinea Pig and Mouse. *Transplantation*, **13**, 541.

SJÖGREN, H. O., HELLSTRÖM, I., BANSAL, S. C. & HELLSTRÖM, K. E. (1971) Suggestive Evidence that the Blocking Antibodies of Tumor Bearing Individuals may be Antigen–Antibody Complexes. *Proc. natn. Acad. Sci. U.S.A.*, **68**, 1372.